# Multidisciplinary and Comparative Investigations of Potential Psychobiotic Effects of *Lactobacillus* Strains Isolated From Newborns and Their Impact on Gut Microbiota and Ileal Transcriptome in a Healthy Murine Model

**DOI:** 10.3389/fcimb.2019.00269

**Published:** 2019-07-25

**Authors:** Bo Ram Beck, Gun-Seok Park, Do Yeun Jeong, Yong Hyun Lee, Sunghoon Im, Won Ho Song, Jihee Kang

**Affiliations:** AtoGen Co., Ltd., Daejeon, South Korea

**Keywords:** psychobiotics, *Lactobacillus reuteri*, anti-inflammation, neurotransmitters, circadian rhythm, gut microbiota, transcriptomics, metagenomics

## Abstract

Psychobiotics are probiotic microorganisms that may exert positive influence on the psychological status of the host. Studies have revealed immunological and microbiological correlations of gut microbiota and the gut-brain axis, and have investigated psychobiotics based on the findings of the gut-brain axis. Considering their mode of actions, the present study sets anti-inflammatory effect, neurotransmitter modulation, and gut microbiota modulation as three essential criteria to evaluate *Lactobacillus casei* ATG-F1 (F1), *L. reuteri* ATG-F3 (F3), and *L. reuteri* ATG-F4 (F4) isolated from newborns as psychobiotics candidates in a healthy mouse model and compares the results with a non-treated control group and an ampicillin-induced gut dysbiosis (Amp) group as a negative control. The F3 and F4 strains showed anti-inflammatory effects *in vitro* in RAW264.7 murine macrophages, and the level of anti-inflammatory cytokine interleukin (IL)-10 increased in ileums of mice orally administered with the F4 strain. Serum dopamine level significantly increased only in the F4-treated group as compared with the control group. Serum serotonin level was unaffected in *Lactobacillus*-treated groups, while a significant decrease in serum serotonin level was observed in the Amp group. Bacteroidetes population increased in fecal samples of the F4-treated group as compared with the control, and *Bacteroidales* S24-7 and *Prevotellaceae* population significantly increased at family level in fecal samples from the F4-treated group as compared with the control. In contrast, the Amp group showed an increase in the level of Proteobacteria and a decrease in the level of Bacteroidetes as compared with the control group. Transcriptome analysis revealed a distinctive clustering in ileums from the F4-treated group as compared to other experimental groups. In addition, the circadian rhythm pathway showed maximum enrichment in ileums of *Lactobacillus*-treated mice, and the F4-treated group showed the highest fold changes in circadian rhythm-related genes (*Dbp, Per1, Per2*, and *Per3*). Conclusively, *L. reuteri* ATG-F4 is suggested as a potential psychobiotics through demonstrations of anti-inflammatory effects, serum dopamine modulation, and gut microbiota modulation in a healthy murine model in the present study. Moreover, we carefully suggest gut circadian rhythm modulation as another important criterion of psychobiotics, which may have an important role in the gut-brain axis.

## Introduction

*Mens sana in corpore sano*, a Latin phrase that means a healthy mind in a healthy body, implies that the physical and psychological systems are closely linked together. In other words, the health status of the body influences the mental status. Recent studies on the microbiota-gut-brain axis revealed the interaction, correlation, and association of the gut microbiota with the mental status of the host (Lyte and Cryan, 2014; Johnson and Foster, 2018). Considering the gastrointestinal tract, the gut health status including the gut microbiota may alter the host's mind, as the gut microbiota is described as the third organ (O'hara and Shanahan, 2006; Guinane and Cotter, 2013). Among the members of the native and acquired inhabitants of the gut microbiota, certain psychotropic bacteria are defined as psychobiotics by Dinan et al. these are the probiotics that influence and benefit the mental health of the host (Dinan et al., 2013).

The impact of gut microbiota on the gut-brain axis has been actively studied in microbiology. The association of gut microbiota with unstable mental health or disorders such as anxiety, depression (Foster and Neufeld, 2013), stress susceptibility (De Palma et al., 2014), autism spectrum disorder (Mayer et al., 2014; Li and Zhou, 2016; Kelly et al., 2017), schizophrenia (Dinan et al., 2014; Nemani et al., 2015; Kelly et al., 2017), Parkinson's disease (Mulak and Bonaz, 2015; Houser and Tansey, 2017), and Alzheimer's disease (Bhattacharjee and Lukiw, 2013; Pistollato et al., 2016; Jiang et al., 2017) is widely discussed in the past decade. The gut-brain axis was thought to be bidirectional, from the gut to the brain and from the brain to the gut (Carabotti et al., 2015). Thus, the gut microbiota-gut-brain axis is a very intriguing topic in the investigation of the host-microbiota interaction. One of goals of the researchers in this field is to improve the mental health status of the host through the modulation of microbiota or certain microbial supplementation that gives rise to the concept of psychobiotics.

To understand the mechanisms underlying the action of psychobiotics, several studies have suggested the various modes of actions of the gut-brain axis from immunological, microbiological, and/or psychological perspectives. The interaction between gut microbiota and gut-brain axis may involve immunological mode of actions such as the balance between proinflammatory and anti-inflammatory responses, modulation of cytokine production in the gut, and crosstalk of the immune cells such as dendritic cells, macrophages, and T and B cells responding to commensal or allogenic microorganisms in the gut (Fung et al., 2017; Bambury et al., 2018). Microbial community factors influence the gut-brain axis through Bacteroidetes present in the gut microbiota, Bacteroidetes/Firmicutes ratio, or changes in Proteobacteria population (Dinan and Cryan, 2015; Fung et al., 2017; Kelly et al., 2017). In addition, microbial metabolites such as short-chain fatty acids produced from microbial fermentation (Bambury et al., 2018; Ho et al., 2018) and direct production of neurotransmitters (e.g., dopamine, serotonin, and γ-amino butyric acid) by microorganisms in the gut may contribute to the gut-brain axis modulation (Cryan and Dinan, 2012; Johnson and Foster, 2018).

Based on the findings and hypotheses of previous studies on the gut-brain axis, herein we set three criteria of potential psychobiotic properties, including anti-inflammatory potentials, influence on neurotransmitters, and modulation of gut microbiota, to develop a procedure to evaluate psychobiotics. By focusing on these criteria, we isolated three *Lactobacillus* species from new born infants, namely, *L. casei* ATG-F1 (F1), *L. retueri* ATG-F3 (F3), and *L. reuteri* ATG-F4 (F4), and investigated their potential psychobiotic properties.

## Methods and Materials

### Bioethics Declaration

Ethics approval for animal study was provided by the Institutional Animal Care and Use Committee (IACUC) of AtoGen Co., Ltd., registration number AEC-20181102-0001 from the Animal and Plant Quarantine Agency of South Korea, approval number ATG-IACUC-REV-180810. Animal care and ethics were conducted as per the guidelines of Animal and Plant Quarantine Agency and Ministry of Food and Drug Safety of South Korea.

### Animals and Experimental Groups

Four-week-old C57BL/6J male mice were purchased from Central Lab Animal Inc., Seoul. Male mice used to avoid any effects of oestrous cycle and related hormonal changes of female mice. Mice were acclimatized for a week in the laboratory and maintained in a controlled environment with a temperature of 23 ± 0.8°C, humidity of 55 ± 3%, and 12 h day/night cycle. Five mice were placed in Sealsafe NEXT individual ventilation cages (Tecniplast, Italy) and fed *ad libitum* with normal diet for rodents (Cargill Inc., Purina®, Korea). After acclimatization, the control mice received distilled water (DW), while the mice from the Amp group received DW containing 1 g/L ampicillin to induce gut microbiota dysbiosis (Le Bastard et al., 2018). Mice from the F1, F3, and F4 groups received DW containing ~1.0 × 10^7^ CFU/mL of each *Lactobacillus* strain as drinking water for 4 weeks. Briefly, *Lactobacillus* strains were overnight cultured in MRS broth for overnight at 37°C, and bacterial cell pellets were harvested by centrifugation at 12,000 × *g*. Resulting bacterial cell pellets were washed 3 times with DW and suspended to drinking water of each *Lactobacillus* treatment group. Resulting acidity of lactobacilli suspended DW was ~ pH 7.0. Initial concentrations of lactobacilli were maintained close to the initial concentration at maximum of 3 days at 23 ± 0.8°C (Figure S1), and the drinking water containing each *Lactobacillus* strain was changed to new sets of drinking water in 3 days intervals, respectively. Weight, food consumption amount, and water consumption amount were monitored throughout the experiments. At experimental endpoint, fresh fecal samples were collected by placing each mouse in an empty cage for 30 to 45 min. Fecal samples of mice were collected at BSL-2 level with an aid of AIRSTREAM® class II biohazard safety cabinet (ESCO Micro Pte Ltd. Singapore). Serum and ileum samples of mice were collected after anesthetization by diethyl ether. Blood samples for serum collection were collected by heart puncture from each mouse, and freshly obtained serum samples were immediately processed for neurotransmitter ELISA assays to avoid oxidation of neurotransmitters. Mice were sacrificed at 3 h mark of the day cycle. Induction of gut dysbiosis owing to ampicillin treatment was confirmed by the enlargement of cecum of mice from the Amp group (Figure S2). Total of two independent sets of animal experiments were performed (total *n* = 10 per experimental group).

### *Lactobacillus* Strains

Two anonymous donors of Daejeon, South Korea, kindly provided fecal samples of newborn babies. *L. casei* ATG-F1 was isolated in January 2016 from the first donor, and *L. reuteri* ATG-F3 and *L. reuteri* ATG-F4 were isolated in June 2018 from the second donor respectively. Fecal samples were opened and processed in BSL-2 AIRSTREAM® class II biohazard safety cabinet for isolation of *Lactobacillus* strains. Each *Lactobacillus* strain was identified through 16S rRNA sequencing. *L. casei* ATG-F1 was used for interspecies comparison and *L. reuteri* strains, for intraspecies comparison. *Lactobacillus* strains were cultured on de Man Rogosa Sharpe (MRS) medium (Difco Laboratories, USA) at 37°C.

### Anti-inflammatory Effects of *Lactobacillus* Strains

#### *In vitro* Anti-inflammatory Effects of Lactobacillus Strains in Murine Macrophage Model

Murine macrophage RAW264.7 cells were cultured up to ~80% confluency in complete Dulbecco's modified Eagle's medium (DMEM, Gibco, USA) supplemented with 10% fetal bovine serum (Gibco, USA) and 1% antibiotic cocktail (penicillin/streptomycin, Sigma-Aldrich, Germany) at 37°C in 5% CO_2_. The cells were collected and seeded at a density of 1 × 10^6^ cells/well in a 24-well cell culture plate (SPL Life Science, Korea) for 24 h. Prior to treatment, *Lactobacillus* strains were lysed by treating the bacterial pellets with lysozyme and sonication, and the lysates were heated at 80°C for 30 min to avoid bacterial growth. About 1 μg/mL of LPS (Sigma-Aldrich, Germany), 100 μg/mL of bacterial lysate, or the combination of LPS and lysate was used treated to cells. Culture supernatants were collected after 24 h of incubation, and IL-10 concentration was determined with IL-10 Quantikine enzyme-linked immunosorbent assay (ELISA) Kit (R&D systems, USA). ELISA results were read with Epoch microplate spectrophotometer (BioTek instruments, USA) at 450 nm wavelength. The inhibition of LPS-induced nitrite production by each lysate was measured by incubating 100 μL of cell culture supernatant and 100 μL of Griess reagent (Sigma-Aldrich, USA) for 10 min at 25°C. The results were analyzed at 540 nm wavelength.

#### Ileum IL-10 Measurement in Murine Models

Ileum tissue samples weighing 100 mg were homogenized in 400 μL of T-PER™ tissue protein extraction reagent (ThermoFisher Scientific, USA) supplied with cOmplete™ mini EDTA-free protease inhibitor cocktail (Merck, Germany) using a hand-held homogenizer (T10 Basic, IKA®, China). Lysates were centrifuged at 10,000 × *g* for 20 min, and the resulting supernatants were analyzed with IL-10 Quantikine ELISA Kit (R&D systems, USA) to measure IL-10 concentration.

### Serum Dopamine and Serotonin Measurement

For serum dopamine level measurement, 200 μL of serum samples were mixed 100 μL of 1 × phosphate-buffered saline (PBS) to adjust the dopamine extraction reaction volume, and subjected to the extraction procedures provided in dopamine ELISA kit (Abnova, Taiwan). For serum serotonin measurement, serum samples were diluted at 1:5 and 1:10 ratios and used with serotonin ELISA kit (Abcam, USA). Competitive ELISA experiments were performed following the manufacturer's instructions.

### Fecal Microbiota Analysis

Genomic DNA extraction from fecal samples was performed using the PowerFecal DNA Isolation Kit (Mo Bio Laboratories, USA). The quantity and quality of extracted DNA were measured using NanoDrop1000 spectrophotometer (Thermo Fisher Scientific, USA) and agarose gel electrophoresis, respectively. The V3–V4 hypervariable regions of the bacterial 16S rRNA were amplified with unique 8 bp barcodes and sequenced on the Illumina MiSeq PE300 platform according to standard protocol (Caporaso et al., 2012). The raw sequence data were submitted to the NCBI's SRA database (NCBI BioProject PRJNA516311). Raw reads were analyzed using the QIIME pipeline (Caporaso et al., 2010). Sequences were quality filtered and clustered into operational taxonomic units at 97% sequence identity according to the SILVA 128 database (Yilmaz et al., 2014). The operational taxonomy units were identified at phylum to genus levels. The unweighted and weighted UniFrac distances were previously obtained and used for PCoA (Lozupone et al., 2010).

### Transcriptome Analysis

Three randomly selected ileum samples from each experimental group of the first animal experiment set were stabilized and preserved in RNAlater solution (Invitrogen, USA) and sent to Macrogen Inc., Korea, library preparation (TruSeq RNA Sample Prep Kit v2, Illumina, USA). RNA sequencing (100 bp, NovaSeq 6000 S4 Reagent Kit, Illumina, USA) was performed to produce raw data (Martin and Wang, 2011). The resulting RNA sequencing results were quality checked using FastQC and trimmed (Illumina adapters) through Trimmomatic (Bolger et al., 2014). All sequences were mapped using HISAT2 (Pertea et al., 2016) and analyzed using SAM tools v1.9 to produce a list of significant differentially expressed genes (DEGs, statistical significance cut-off *p* < 0.05) as compared with the control group. Gene ontology (GO) enrichment analysis of significant DEGs of each experimental group compared to the control group was performed using database of Gene Ontology (http://geneontology.org/). Kyoto Encyclopedia of Genes and Genomes (KEGG) database was used for pathway enrichment analysis (Altermann and Klaenhammer, 2005; Kanehisa et al., 2016). Based on the DEG result, the genes of interest were selected and a heatmap was generated using online tool Morpheus (Broad Institute, https://software.broadinstitute.org/morpheus/). The raw sequencing data produced in the present study were deposited in NCBI's SRA database (NCBI BioProject PRJNA52169).

### Statistics

One-way analysis of variance (ANOVA) with Tukey's *post-hoc* test was performed to compare multiple columns. Unpaired two-tailed *t*-test was used to separately compare two independent groups. For statistical analysis of transcriptome results, absolute values of transcript fold changes equal or larger than 2.0 were processed with independent *t*-tests. Gene-set enrichment analysis was performed through the modified fisher's exact test to analyze significances of KEGG pathways generated based on transcriptome results.

## Results

### Anti-inflammatory Effects of *Lactobacillus* Strains

**Figure 1:**
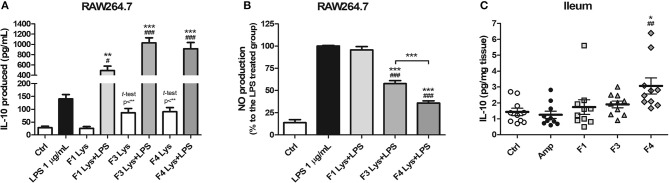
Anti-inflammatory effects of *Lactobacillus casei* ATG-F1 (F1), *L. reuteri* ATG-F3 (F3), and *L. reuteri* ATG-F4 (F4). **(A)** *in vitro* IL-10 production was determined in murine macrophage RAW264.7 cells treated with 100 μg/mL of each *Lactobacillus* lysate with or without LPS co-treatment. **(B)** Nitrite reduction in the LPS co-treated groups. Results of three independent experiments of triplicate wells are shown as mean ± SEM. **(C)** *in vivo* IL-10 concentration in the ileum was determined after the oral administration of lactobacilli and compared with the control (Ctrl) and ampicillin-induced gut dysbiosis (Amp) groups after 4 weeks of the experiment in healthy C57BL/6J mice. Each dot represents one mouse (*n* = 10 per each group). For **(A)** and **(B)**, statistical significance was as follows: ^*^p < 0.05, ^**^p < 0.01, ^***^p < 0.0001 vs. the Ctrl group; **(A)** ^#^*p* < 0.05, ^*###*^*p* < 0.0001 vs. the LPS group; **(B)** ^***^p < 0.0001 vs. the LPS group; **(B)** ^*###*^*p* < 0.0001 vs. F1 group. For **(C)**, statistical significance was compared to the Ctrl (^*^p < 0.05) and Amp group (^*##*^*p* < 0.01). Statistics were determined with one-way ANOVA along with Tukey's *post-hoc* test for all experiments, and additional *t*-test was used in **(A)** to separately compare the Ctrl group and *Lactobacillus* lysate-treated without LPS treatment groups.

As a mode of action, the anti-inflammatory potentials of *Lactobacillus* strains were evaluated. The concentration of the anti-inflammatory cytokine interleukin (IL)-10 was elevated following co-treatment of RAW264.7 cells with lysates of all the three strains and lipopolysaccharide (LPS) as compared to treatment with LPS only (Figure 1A). In comparison with the control group, the F3 and F4 lysates induced higher production of IL-10 by RAW264.7 cells following a single treatment of each lysate without LPS co-treatment (*p* < 0.01, determined by *t*-test). LPS-induced nitrite production by RAW264.7 cell was significantly reduced following treatment with the F3 and F4 lysates, while treatment with the F1 lysate had no effect on nitrite production. The F4 lysate treatment showed the highest reduction in nitrite level among all treatment groups (Figure 1B). In animal experiments, IL-10 concentration significantly increased in the ileums of the F4-administrated group as compared with ileums from the control group and the ampicillin-induced gut dysbiosis (Amp) group at the end of 4 weeks of *Lactobacillus* administration (Figure 1C). Taken together, only the F4 strain showed anti-inflammatory potentials in both *in vitro* and *in vivo* experiments.

### Influence of Lactobacilli on the Levels of Circulatory Dopamine and Serotonin

**Figure 2:**
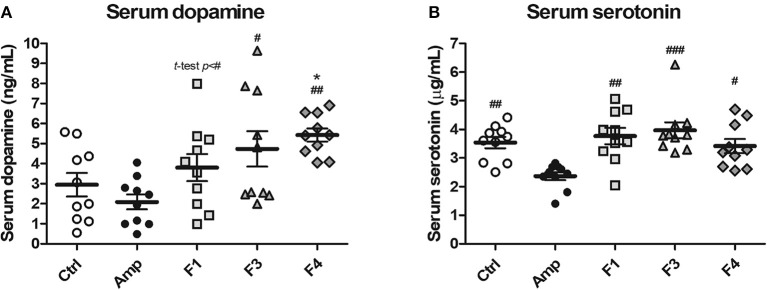
Serum dopamine and serotonin modulation by oral administration of *Lactobacillus* strains for 4 weeks in healthy C57BL/6J mice. Serum **(A)** dopamine and **(B)** serotonin concentration in the groups treated with *Lactobacillus casei* ATG-F1 (F1), *L. reuteri* ATG-F3 (F3), and *L. reuteri* ATG-F4 (F4) as compared to the control group (Ctrl) and ampicillin-induced gut dysbiosis (Amp) group. Each dot represents one mouse (*n* = 10 each group). Statistical significance was as follows: ^*^p < 0.05 vs. the Ctrl group; ^#^*p* < 0.05, ^*##*^*p* < 0.01, ^*###*^*p* < 0.0001 vs. the Amp group. Statistics were determined with one-way ANOVA along with Tukey's *post-hoc* test for all experiments, while additional *t*-test was performed in **(A)** to separately compare F1 and Amp groups.

To examine the effects of lactobacilli on circulatory neurotransmitters, serum dopamine and serotonin levels were measured from the serum samples of each experimental group at the end of 4 weeks from each *Lactobacillus* oral administration. A significant increase in serum dopamine concentration was observed in the F4-treated group as compared with the control and Amp groups. The F1-treated and F3-treated groups showed an increase in the level of serum dopamine as compared with Amp group (Figure 2A). However, serum dopamine levels showed variations in the F1 and F3 groups and exhibited no statistical significance as compared with the control group. A minor decrease in serum dopamine level was observed in the Amp group that was not significantly different as compared with control group. Serum serotonin level was unaffected following oral treatment with *Lactobacillus* strains as compared with the control group; however, a significant decrease in serum serotonin level was observed in the Amp group as compared to other experimental groups (Figure 2B). These results suggest that the oral microbial supplementation or gut microbiota disruption may influence the levels of circulatory neurotransmitters of the host.

### Modulation of Gut Microbiota by Lactobacilli

**Figure 3:**
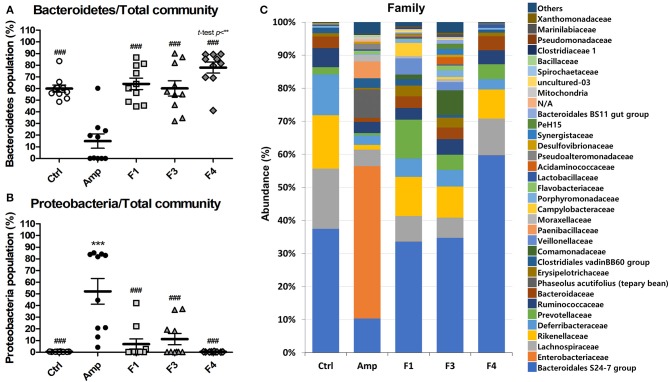
Changes in fecal bacterial community in the ampicillin-induced gut dysbiosis group (Amp) and the groups treated with *Lactobacillus casei* ATG-F1 (F1), *L. reuteri* ATG-F3 (F3), and *L. reuteri* ATG-F4 (F4) as compared to the control (Ctrl) group. Abundance of the phyla **(A)** Bacteroidetes and **(B)** Proteobacteria are shown in dot plot (total read counts). Each dot represents one mouse (*n* = 10 per group). Statistical significance was as follows: ^**^p < 0.01, ^***^p < 0.0001 vs. the Ctrl group; ^*###*^*p* < 0.0001 vs. the Amp group. Statistics were determined with one-way ANOVA along with Tukey's *post-hoc* test, and *t*-test was performed in **(A)** to compare the F4 and Ctrl groups. Top 35 abundant family level taxa in average of 10 mice from each experimental group were shown in **(C)** stacked bar plot.

To examine the effects of lactobacilli on the gut microbiota, the bacterial community from fecal samples of each experimental group was analyzed. The alpha-diversity analysis revealed a significant increase in the population of Bacteroidetes in the F4-treated group as compared to other groups, while the Amp group showed a significant increase in the abundance of Proteobacteria (Figures 3A,B, Figure S3). The families *Bacteroidales* S24-7 (phylum Bacteroidetes) and *Enterobacteriaceae* (phylum Proteobacteria) showed identical patterns of population shifts in the family level community analysis as compared with phylum level analysis (Figure 3C, Figures S4A–C). Only the F4-treated group showed a significant increase in the abundance of the family *Prevotellaceae* (phylum Bacteroidetes) as compared with the control or Amp group, while *Lactobacillaceae* population significantly increased only in the F4-treated group as compared to all other experimental groups (Figures S4B,D).

**Figure 4:**
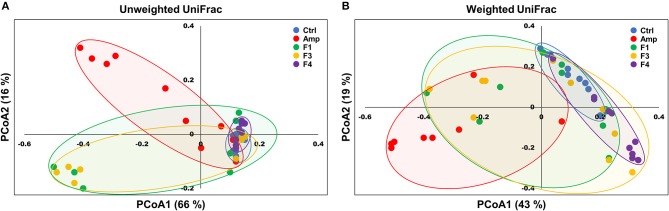
Principal coordinate analysis (PCoA) plots of fecal bacterial communities of the control (Ctrl), ampicillin-induced gut dysbiosis (Amp), *Lactobacillus casei* ATG-F1 (F1)-, *L. reuteri* ATG-F3 (F3)-, and *L. reuteri* ATG-F4 (F4)-treated groups at the end of 4 weeks of oral administration. Plots based on **(A)** unweighted and **(B)** weighted UniFrac distance metrics are shown. Each dot represents one mouse (*n* = 10 per group).

Beta-diversity was examined by the principal coordinate analysis (PCoA) to compare the composition of microbiota using UniFrac distance. Weighted-UniFrac takes into account the relative abundance of species/taxa shared between samples, whereas unweighted-UniFrac only considers presence/absence. The microbiota from the Amp group separated from those of other groups (Figure 4). The composition of microbiota of the F4-treated group was not distinct from that of control group in the unweighted PCoA plot (Figure 4A), whereas the microbial population showed a shift in the F4-treated group, as shown in weighted PCoA plot (Figure 4B). Mirroring the α-diversity differences, there were differences in clustering of *Lactobacillus* treated groups compared to the control group and Amp group. However, the change in the microbial population in the F1- and F3-treated groups was inconsistent as compared to the control and F4-treated groups. In the case of the F1- and F3-treated groups, they resulted with different changes between biological replicates, while the F4 treated group showed relatively similar changes between each mouse in the same group. The microbial community scattering shown in the F1- and F3-treated group may be affected by initial gut microbiota of each mouse, however, the F4 treatment seems to force and maintain the abundance of phylum Bacteroidetes at higher level compared to the control group as shown in weighted PCoA plot (Figure 4B).

### Transcriptional Modulation by Lactobacilli in the Ileum of Mice

**Figure 5:**
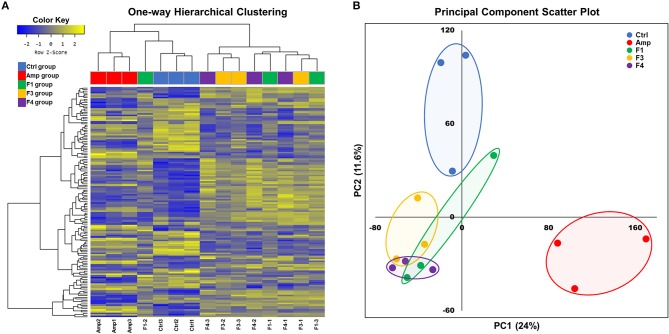
Overall transcriptome analysis results. **(A)** Hierarchical clustering of the gene with significant changes (142 genes, |fold change| ≥2, *p* < 0.05 determined by independent *t*-test), and **(B)** principal component scatter plot of transcriptomes of the ileum samples from each experimental group. Experimental groups (*n* = 3 per group) shown in plots are as follows: Ctrl, the control group; Amp, the ampicillin-induced gut dysbiosis group; F1, *Lactobacillus casei* ATG-F1-treated group; F3, *L. reuteri* ATG-F3-treated group; F4, *L. reuteri* ATG-F4-treated group.

Overall transcriptome one-way hierarchical clustering analysis revealed three distinct clusters, namely the control, Amp, and *Lactobacillus*-treated groups (Figure 5A). Each sample from the F4-treated group showed the closest grouping in the transcriptome among *Lactobacillus*-treated groups in principal component scatter plot analysis (Figure 5B). A total of 142 significant genes with satisfactory cut-off values (|fold change| ≥2, *p* < 0.05) were detected in the experiment; the number of DEGs from each experimental group as compared with the control group are as follows: the Amp group, 61 genes; the F1-treated group, 37 genes; the F3-treated group, 45 genes; the F4-treated group, 84 genes (Figure S5).

**Figure 6:**
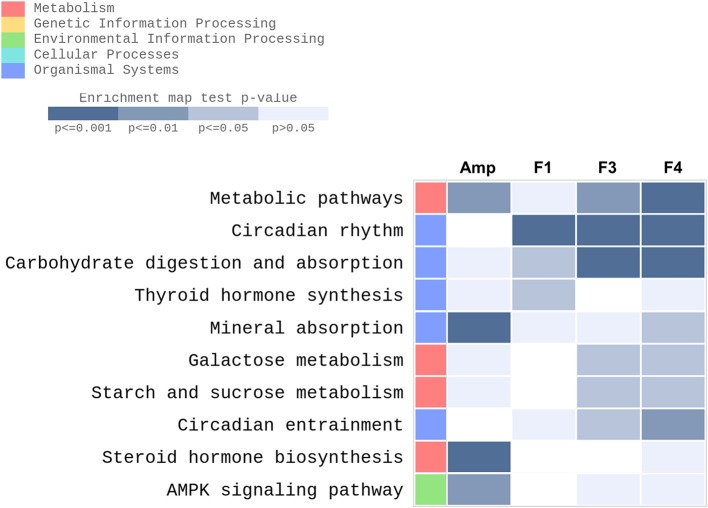
Top 10 KEGG enriched pathways determined in the ileum of experimental groups (*n* = 3 per group) with gene-set enrichment analysis through modified fisher's exact test as compared with the control group were plotted as a heatmap. Experimental groups shown in the heatmap are as follows: Amp, the ampicillin-induced gut dysbiosis group; F1, *Lactobacillus casei* ATG-F1-treated group; F3, *L. reuteri* ATG-F3-treated group; F4, *L. reuteri* ATG-F4-treated group.

KEGG pathway enrichment was performed using the data set generated from transcriptome analysis of ileum tissue samples. GO enrichment analysis results are provided as Supplementary Materials (Figure S6, Tables S1–S4). Metabolic pathways, circadian rhythm, carbohydrate digestion and absorption, thyroid hormone synthesis, mineral absorption, galactose metabolism, starch and sucrose metabolism, circadian entrainment, steroid hormone biosynthesis, and 5′-AMP-activated protein kinase signaling pathway were identified as the top 10 KEGG enriched pathways in the ileum of *Lactobacillus*-treated groups after excluding disease and infection model pathways (Figure 6). Transcriptional modulations of circadian rhythm and circadian entrainment were affected following *Lactobacillus* treatments as compared with the control group, while the Amp group showed no significant response in these two categories.

**Figure 7:**
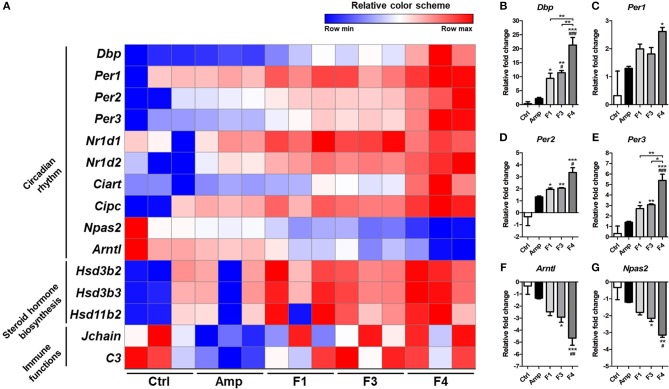
**(A)** Heatmap of 15 selected genes based on KEGG pathway enrichment shown as a relative color scheme for each row. **(B–G)** Significant relative fold changes in circadian rhythm-associated genes plotted as bar graphs. Experimental groups (*n* = 3 per group) shown in the heatmap and bar graphs are as follows: Ctrl, the control group; Amp, the ampicillin-induced gut dysbiosis group; F1, *Lactobacillus casei* ATG-F1-treated group; F3, *L. reuteri* ATG-F3-treated group; F4, *L. reuteri* ATG-F4-treated group. For the graph of relative fold change, statistical significance was tested with one-way ANOVA along with Tukey's *post-hoc* test as follows: ^*^p < 0.05. ^**^p < 0.01, ^***^p < 0.0001 vs. Ctrl group; ^#^*p* < 0.05, ^*##*^*p* < 0.01, ^*###*^*p* < 0.0001 vs. Amp group.

Based on the KEGG pathway enrichment analysis, 15 significantly modulated gene sets that may relate to the alteration of mental status and gut dysbiosis were plotted as a heatmap (Figure 7A). Circadian rhythm-related genes (*Dbp, Per1, Per2, Per3, Nrld1, Nrld2, Ciart, Cipc, Npas2*, and *Arntl*) and steroid hormone biosynthesis-related genes (*Hsd3b2, Hsd3b3*, and *Hsd11b2*) were significantly modulated by all *Lactobacillus* strains, and the strongest degree of transcriptional modulation was observed in the F4-treated group. No significant modulation of immune functions was observed among the control and *Lactobacillus*-treated groups, but the Amp group showed a significant decrease in *Jchain* and *C3* genes in transcriptome analysis. Among circadian rhythm-related genes, *Dbp* expression was notably increased in *Lactobacillus*-treated groups as compared to the control and Amp groups, and the F4-treated group showed the highest fold increase as compared to the F1- and F3-treated groups. Correlation was reported in the expression patterns between *Dbp* and circadian rhythm genes (Figures 7B–G). Circadian rhythm genes *Per1, Per2, Per3* (Figures 7C–E) showed a negative correlation with *Arntl* and *Npas2* (Figures 7F,G), the negatively regulated genes in the circadian rhythm pathway.

## Discussion

IL-10, a key cytokine, plays an important role in immune regulation and suppression of inflammation and is produced by dendritic cells, macrophages, and regulatory T cells following pathological inflammatory immune responses (Saraiva and O'garra, 2010; Ouyang et al., 2011). Circulating LPS is one of the important pathogenic factors in neuroinflammation in the host body (Qin et al., 2007). Circulating LPS is derived from gram-negative bacteria from the gut during gut barrier dysfunction, a condition termed as “leaky gut” owing to chronic inflammation (Maes et al., 2008). This phenomenon negatively influences the mind status of the host through the gut-brain axis through neuroinflammation (Wang et al., 2010). The establishment of an immunological status potentiated to anti-inflammation which is described as a regulatory immune tone by Beck et al. (2016) in the gut as a response to probiotics may decrease the permeability of the gut and prevent “leaky gut.” Based on contributions of anti-inflammatory effects on neuroprotection, several studies demonstrated neuroprotective effects of lactobacilli or lactobacilli-fermented products (Musa et al., 2017; Yim et al., 2018; Wang et al., 2019). Therefore, the anti-inflammatory potentials of the F4 strain both *in vitro* IL-10 stimulation and inhibition of NO production in murine macrophage cell model and increased IL-10 concentration in the ileums of F4-treated group may suggest the mode of actions of this potential psychobiotic bacterium, as evident from the well-reviewed interactions between the immune and nervous system through the gut microbiota by Fung et al. (2017).

As a neurotransmitter and neuromodulator, dopamine plays an important role in tuning the psychological status of the host in several psychosis symptoms (Dobryakova et al., 2015; Ayano, 2016). For instance, a decrease in dopamine level was associated with attention deficit hyperactivity disorder and Parkinson's disease (Ayano, 2016), while any increase in the level of circulatory dopamine, as seen following the F4 treatment, may contribute to the prevention of such psychosis. Consistent with the result of transcriptomics, the expression of the genes related to steroid hormone biosynthesis (*Hsd3b2, Hsd3b3*, and *Hsd11b2*) increased following the F4 treatment; Hsd11b2 enzyme (11-β-hydroxysteroid dehydrogenase 2) is involved in the conversion of cortisol to cortisone (Quinkler and Stewart, 2003). The increase in the expression of *Hsd11b2* may contribute to stress tolerance and dopamine overexpression via cortisol reduction, wherein neurotransmitters and cortisol show a negative correlation as demonstrated in other studies (Field et al., 2005; Issa et al., 2010). Although the association between mental safety and observed increment in dopamine level by the F4 strain needs to be confirmed in further studies, no symptoms of possible psychosis, such as anxiety, abnormal behaviors, self-mutilation, or physiological changes (body weight, amount of feed and water consumption), were observed in the present study as a response to increased dopamine level (Figure S7). In contrast, the level of serotonin was unaffected by *Lactobacillus* treatment; however, a significant decrease in serum serotonin level was reported in the Amp group, suggesting that gut dysbiosis may have contributed to the development of psychosis because serotonin is an important factor related to mood and cognition (Jenkins et al., 2016). As per the results of the present study, unlike dopamine, serotonin levels were maintained among the experimental groups during continuous *Lactobacillus* administration except in the Amp group. The increase in serotonin level observed in autism spectrum disorder (Gabriele et al., 2014), and this may indirectly support the safety of serum neurotransmitter modulation following the F4 treatment in psychological aspects, as the serum serotonin level was unaffected. However, the present study evaluated serum circulating dopamine and serotonin which limits their influences to the peripheral system, and yet it is not clear where the source of increased dopamine is. Moreover, neurotransmitters cannot pass through the blood-brain barrier, but the interactions in gut-brain axis are reported numerous times. To understand gut and gut microbiota to brain communication, the vagus nerve is suspected and studied as a route of communication between gut-derived or gut microbiota derived-neurotransmitters (Bercik et al., 2011; Bravo et al., 2011; Perez-Burgos et al., 2012; Bonaz et al., 2018). Nevertheless, serum neurotransmitters are possible core markers of psychobiotics.

The supplementation with probiotic strains may change the intestinal microbiome, but the microbial community shifts following probiotic administration seems to be a strain-specific effect. Although the F3 and F4 strains belong to the same species (*L. reuteri*), their influence on the structure of the microbial community was different. The F1 (*L. casei*) and F3 treatment resulted in similar community shifts in the analysis of microbiota. The F4 treatment induced a significant microbial community shift which resulted in the domination of phylum Bacteroidetes, including *Bacteroidales* S24 group and *Prevotellaceae*, as compared to all other experimental groups. Although modulation of Bacteroidetes population through probiotics administration that links to mental illness or mood disorder is scarce yet, one study reports increased Bacteroidetes population along with mental health improvements after *Bifidobacterium infantis* M-63 intervention in a human trial (Ma et al., 2019). Yet more studies are warranted for the identification of active substances from the F4 strain, it is intriguing that the F4 treatment resulted in a consistent microbial shift in each F4-treated mouse. For example, one study demonstrated that the exopolysaccharides of *L. reuteri* may benefit the growth of *Bacteroides thetaiotaomicron* (Van Bueren et al., 2015). The change in Bacteroidetes population in the gut microbiota correlates with mental health (Dinan and Cryan, 2015; Fung et al., 2017); thus, the F4 strain is a potential psychobiotic bacterium owing to its ability to enhance Bacteroidetes population.

Circadian rhythm or the biological clock that runs at ~24 h intervals, seems to be another factor that may influence the psychological status of the host (Mcclung, 2013). The circadian rhythm compartments such as *Per, Arntl*, or *Npas2* genes are thought to be closely related to mood disorders (Nievergelt et al., 2006; Partonen et al., 2007). In particular, circadian rhythms in the peripheral tissues, or gut in this case, are associated with the immunity, metabolism, and barrier function in the gut (Konturek et al., 2011). Both the host factors as well as the gut microbiota interact with the circadian rhythm of the host (Voigt et al., 2014; Rosselot et al., 2016; Thaiss et al., 2016). *Dbp* is suspected to be related to the circadian oscillation (Stratmann et al., 2010) and its expression was notably increased following *Lactobacillus* treatments; the highest expression level was reported in the F4-treated group. In addition, the strong influence of F4 strain on the circadian rhythm was observed through transcriptomic and KEGG pathway analyses through the upregulation of genes such as *Per1, Per2*, and *Per3* along with *Dbp* (Figure S8). A similar increase in the oscillation of circadian rhythm was reported in a dietary restriction *Drosophila* model study related to increased fat metabolism and life span (Katewa et al., 2016). The present study showed significant enrichment of the metabolic pathway category in KEGG pathway analysis following treatment with the F4 strain. The dysfunction in the circadian rhythm was thought as a risk factor in Parkinson's disease (Lauretti et al., 2017), and recent reports have confirmed the close association between circadian rhythm as well as dopamine and human mood (Radwan et al., 2018). These findings need further validation to reveal the link with the mechanism of psychobiotics in future. Thus, the present study carefully suggests that the circadian rhythm function may be one of important markers and criteria of psychobiotics.

To conclude, the observations such as increased anti-inflammatory potential, upregulated serum dopamine expression, and enhanced *Bacteroidetes* population in *L. reuteri* ATG-F4-treated mice demonstrate the psychobiotic potentials of *L. reuteri* ATG-F4, which showed the highest modulation of intestinal circadian rhythm compartments. The present study also suggests that the psychobiotic effects are species- and strain-specific. Although psychobiotic candidates must be investigated for their effects on various mental illness models and further explored to facilitate the development of effective psychobiotics, we carefully suggest the criteria examined in the present study, including the combination of anti-inflammatory effect, influence on host neurotransmitters, gut microbiota modulation, and circadian rhythm modulation, as effective markers for the screening of the potential psychobiotics to improve mental health.

## Data Availability

The datasets generated and/or analyzed during the current study are available at NCBI's repository. The raw sequence data of bacterial community sequencing are submitted to NCBI SRA database (NCBI BioProject PRJNA516311, https://www.ncbi.nlm.nih.gov/bioproject/?term=PRJNA516311). The sequencing raw data of transcriptome analysis discussed in this publication are deposited at NCBI SRA database (NCBI BioProject PRJNA52169, https://www.ncbi.nlm.nih.gov/bioproject/521649).

## Ethics Statement

Ethics approval for animal study was provided by the Institutional Animal Care and Use Committee (IACUC) of AtoGen Co., Ltd., registration number AEC-20181102-0001 from the Animal and Plant Quarantine Agency of South Korea, approval number ATG-IACUCREV-180810. Animal care and ethics were conducted as per the guidelines of Animal and Plant Quarantine Agency and Ministry of Food and Drug Safety of South Korea.

## Author Contributions

BB conceived and designed experiments, isolated strains F3 and F4, conducted animal experiments, and participated in writing, editing, and the correspondence of the manuscript. G-SP produced and analyzed meta-analyses data and participated in manuscript writing. DJ and YL participated in *in vitro* and *in vivo* assays with cells and animals. SI and WS were involved in the characterization, cultivation, and management of bacterial strains. JK isolated and provided strain F1 and were involved in manuscript editing and correspondence.

### Conflict of Interest Statement

All authors were employed by AtoGen Co., Ltd. The authors declare that this study received no third-party funding, and AtoGen Co., Ltd. supported the present study with non-financial supports.
