# Supplementary material for: Multidisciplinary and Comparative Investigations of Potential Psychobiotic Effects of Lactobacillus Strains Isolated From Newborns and Their Impact on Gut Microbiota and Ileal Transcriptome in a Healthy Murine Model
Source: Front Cell Infect Microbiol. 2019 Jul 25;9:269. doi: 10.3389/fcimb.2019.00269 (PMC6677118; doi:10.3389/fcimb.2019.00269)
Supplement: Supplementary file 1 [file Data_Sheet_1.docx]

Supplementary Material

Multidisciplinary and Comparative Investigations of Potential Psychobiotic Effects of *Lactobacillus* Strains Isolated from Newborns and Their Impact on Gut Microbiota and Ileal Transcriptome in a Healthy Murine Model

**Bo Ram Beck^1^*, Gun-Seok Park^1^, Do Yeun Jeong^1^, Yong Hyun Lee^1^, Sunghoon Im^1^, Won Ho Song, Jihee Kang^1^***

^1^AtoGen Co., Ltd., Daejeon, Republic of Korea

***Correspondence:**

BR Beck

[brbr777@hanmail.net](mailto:brbr777@hanmail.net)

J Kang

[jhkang@atogen.co.kr](mailto:jhkang@atogen.co.kr)

# Supplementary Figures


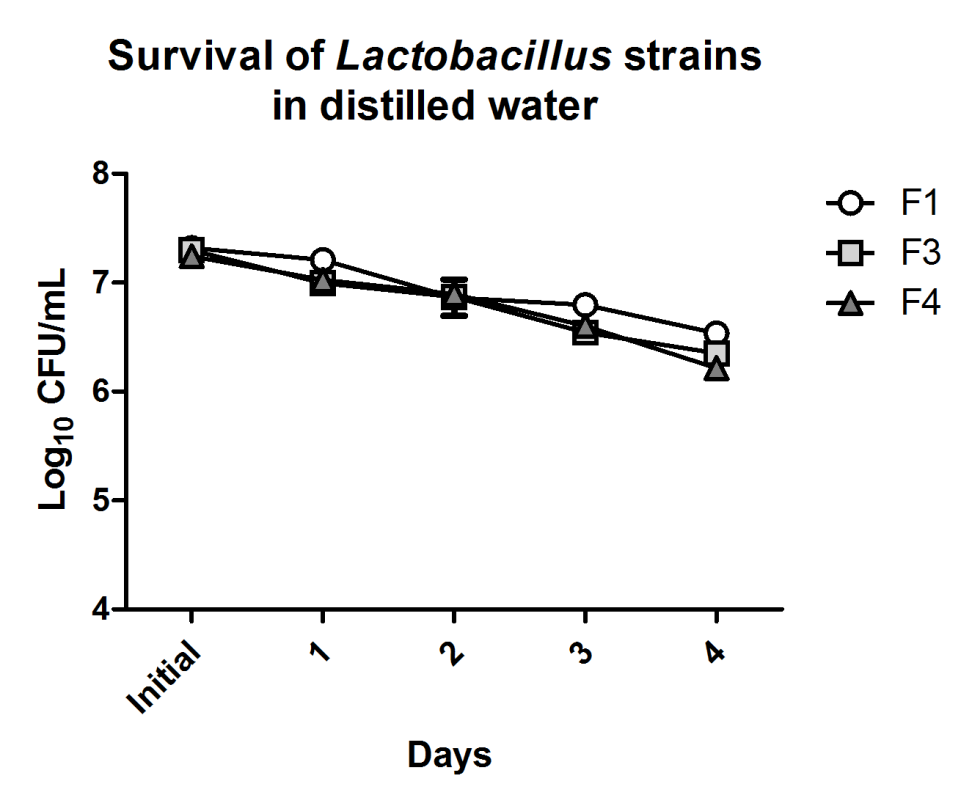


Figure S1. Survival of *Latobacillus* strains in distilled water at 23°C ± 0.8°C. Abbreviations of each *Lactobacillus* strain are as follows; F1, *Lactobacillus casei* ATG-F1; F3, *L. reuteri* ATG-F3; F4, *L. reuteri* ATG-F4.


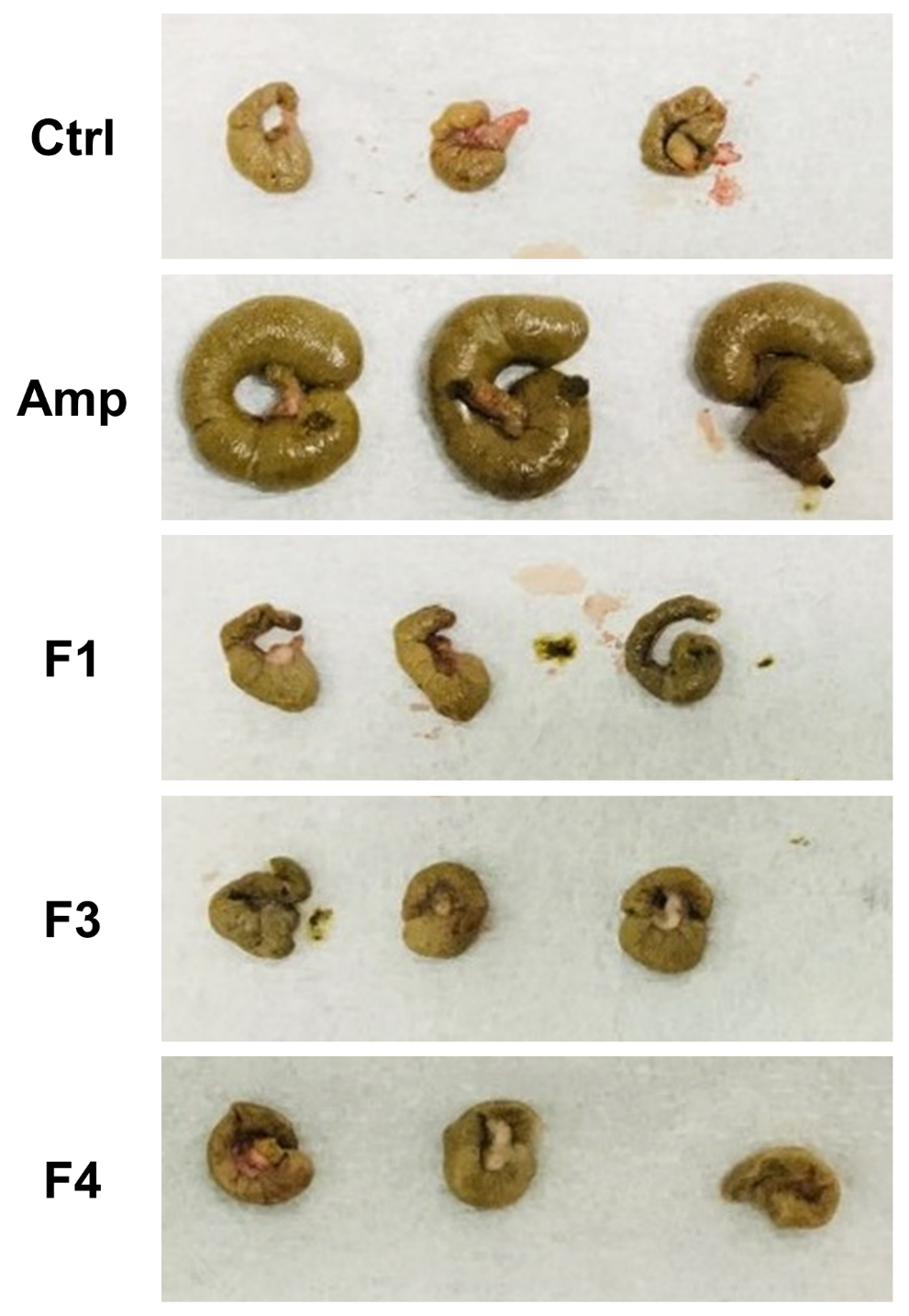


Figure S2. Cecum samples from each experimental group (representative n = 3). Abbreviations of each experimental group are as follows: Ctrl, control group; Amp, ampicillin-induced gut dysbiosis group; F1, *Lactobacillus casei* ATG-F1-treated group; F3, *L. reuteri* ATG-F3-treated group; F4, L. reuteri ATG-F4-treated group.


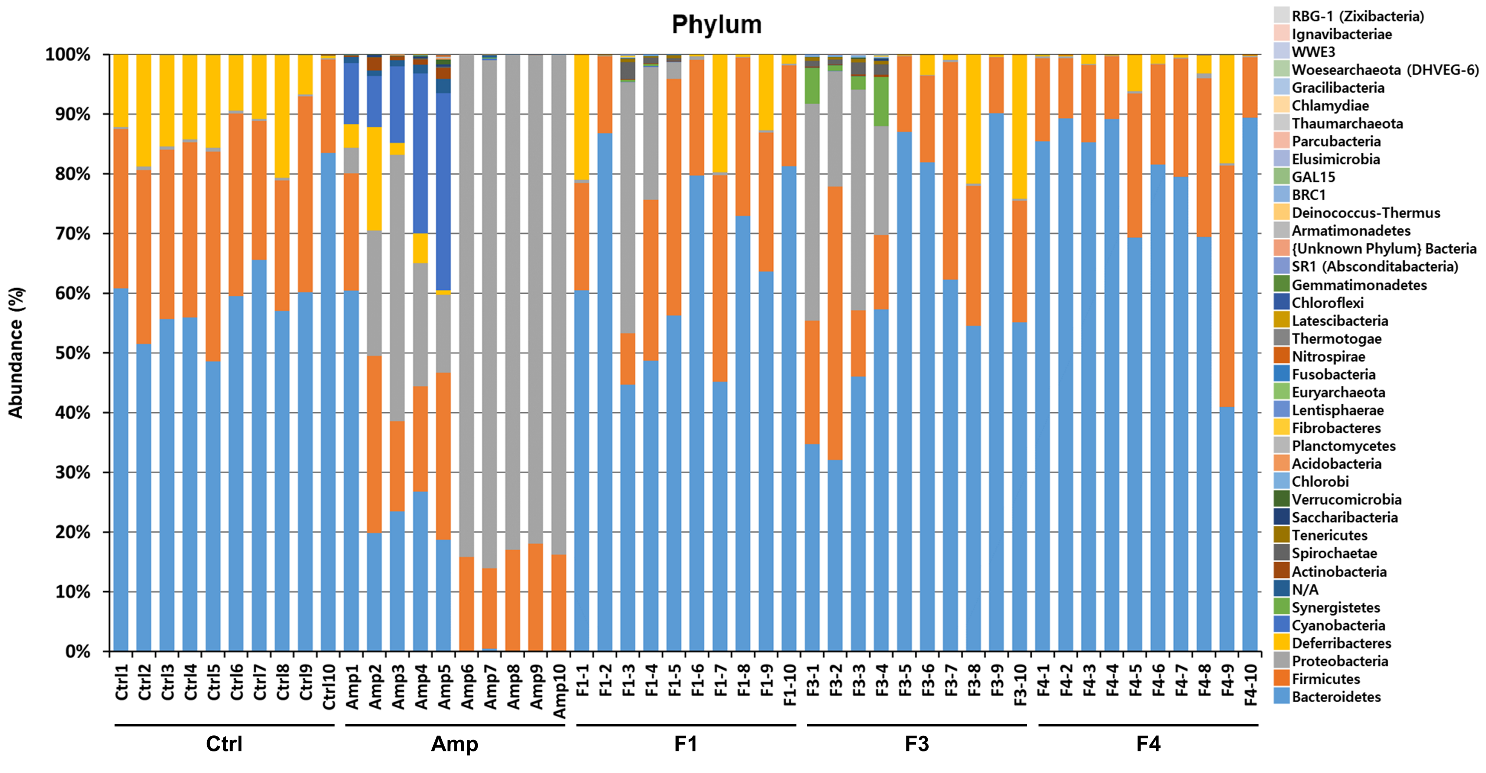


Figure S3. Changes in fecal bacterial community at phylum level in the control group (Ctrl), ampicillin-induced gut dysbiosis group (Amp), and the groups treated with *Lactobacillus casei* ATG-F1 (F1), *L. reuteri* ATG-F3 (F3), and *L. reuteri* ATG-F4 (F4). Each individual bar represents one mouse from each experimental group.


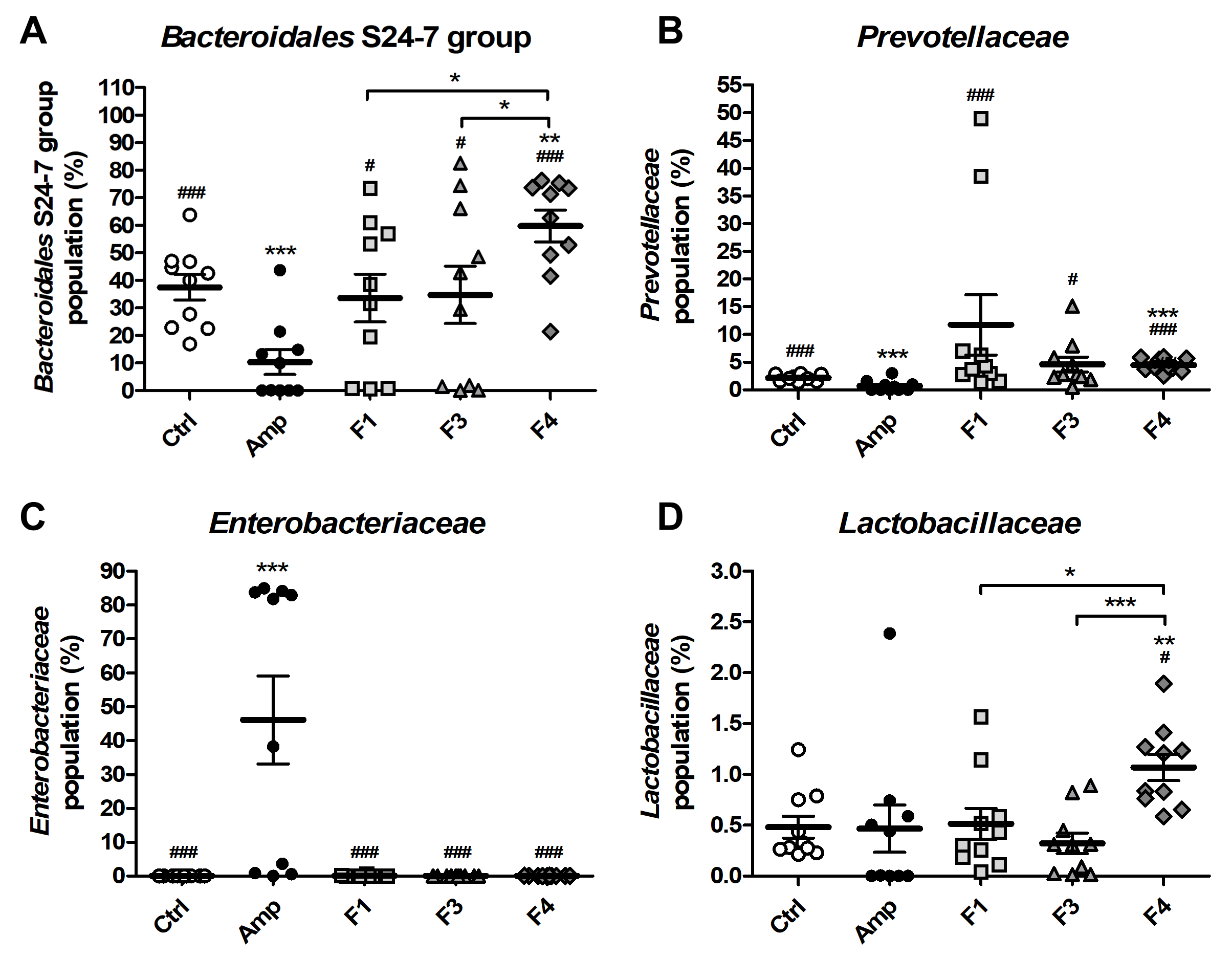


Figure S4. Changes in fecal bacterial community in ampicillin-induced gut dysbiosis group (Amp), *Lactobacillus casei* ATG-F1 (F1)-treated, *L. reuteri* ATG-F3 (F3)-treated, and *L. reuteri* ATG-F4 (F4)-treated groups as compared with the control (Ctrl) group at family level. Abundance of the family (A) *Bacteroidales* S24-7 and (B) *Proteobacteria*, (C) *Enterobacteriaceae*, and (D) *Lactobacillaceae* is shown in the dot plot (total read counts). Each dot represents one mouse (n = 10 per each group). Statistical significance is as follows: **p* < 0.05, ***p* < 0.01, ****p* < 0.0001 versus Ctrl group; #*p* < 0.01, ###*p* < 0.0001 versus Amp group. Directional lines indicate significance between *Lactobacillus*-treated groups. Statistical significance between each group was determined with *t*-test.


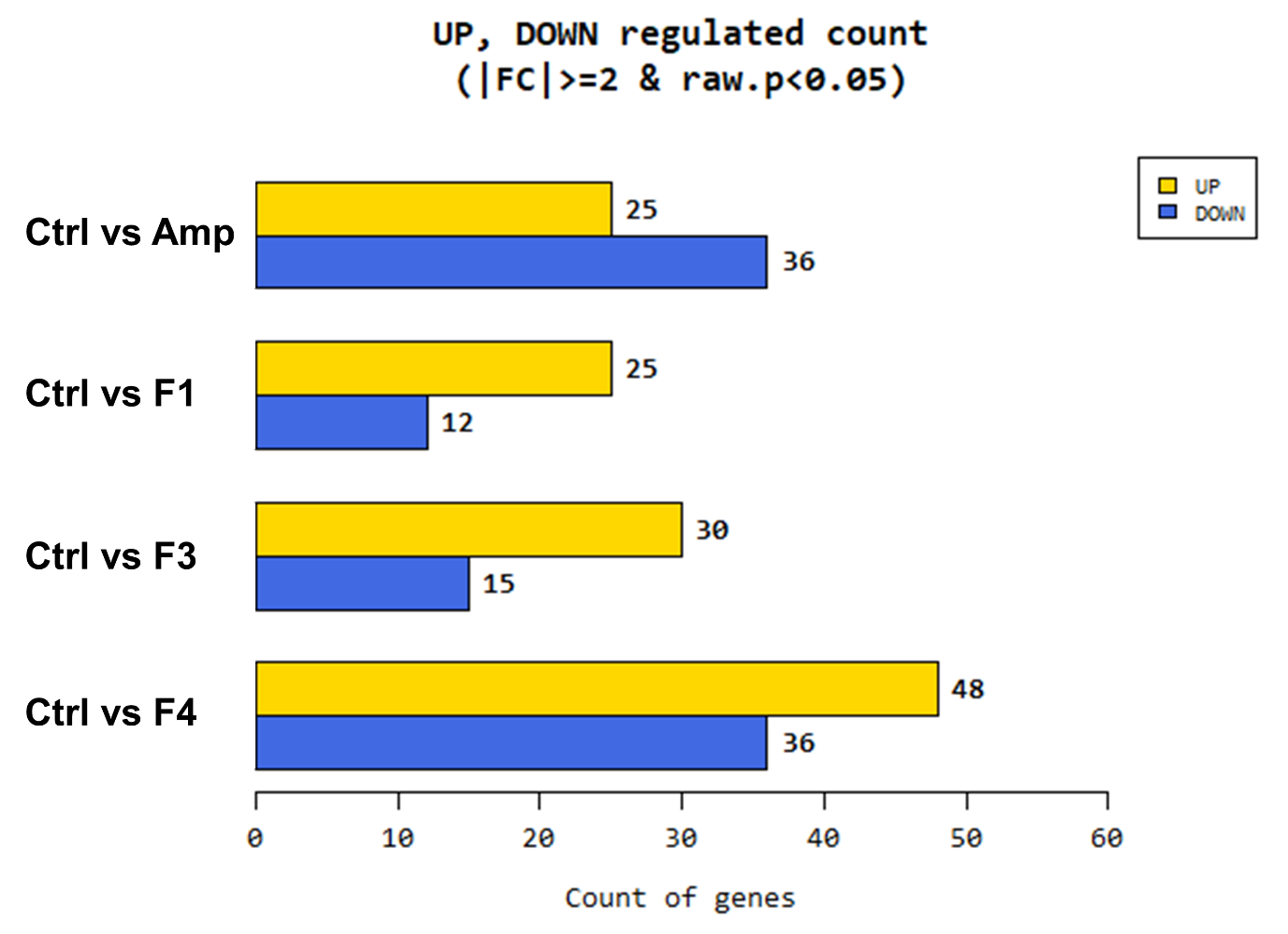


Figure S5. Upregulated and downregulated genes in the transcriptome analysis of the ileum samples from the experimental groups as compared to those from the control group (n = 3 per group). Significant cut-off criteria were |fold change| ≥ 2 and *p* < 0.05, as determined with independent *t*-tests. Experimental groups shown in the graph are as follows: Ctrl, control group; Amp, ampicillin-induced gut dysbiosis group; F1, *Lactobacillus casei* ATG-F1-treated group; F3, *L. reuteri* ATG-F3-treated group; F4, *L. reuteri* ATG-F4-treated group.


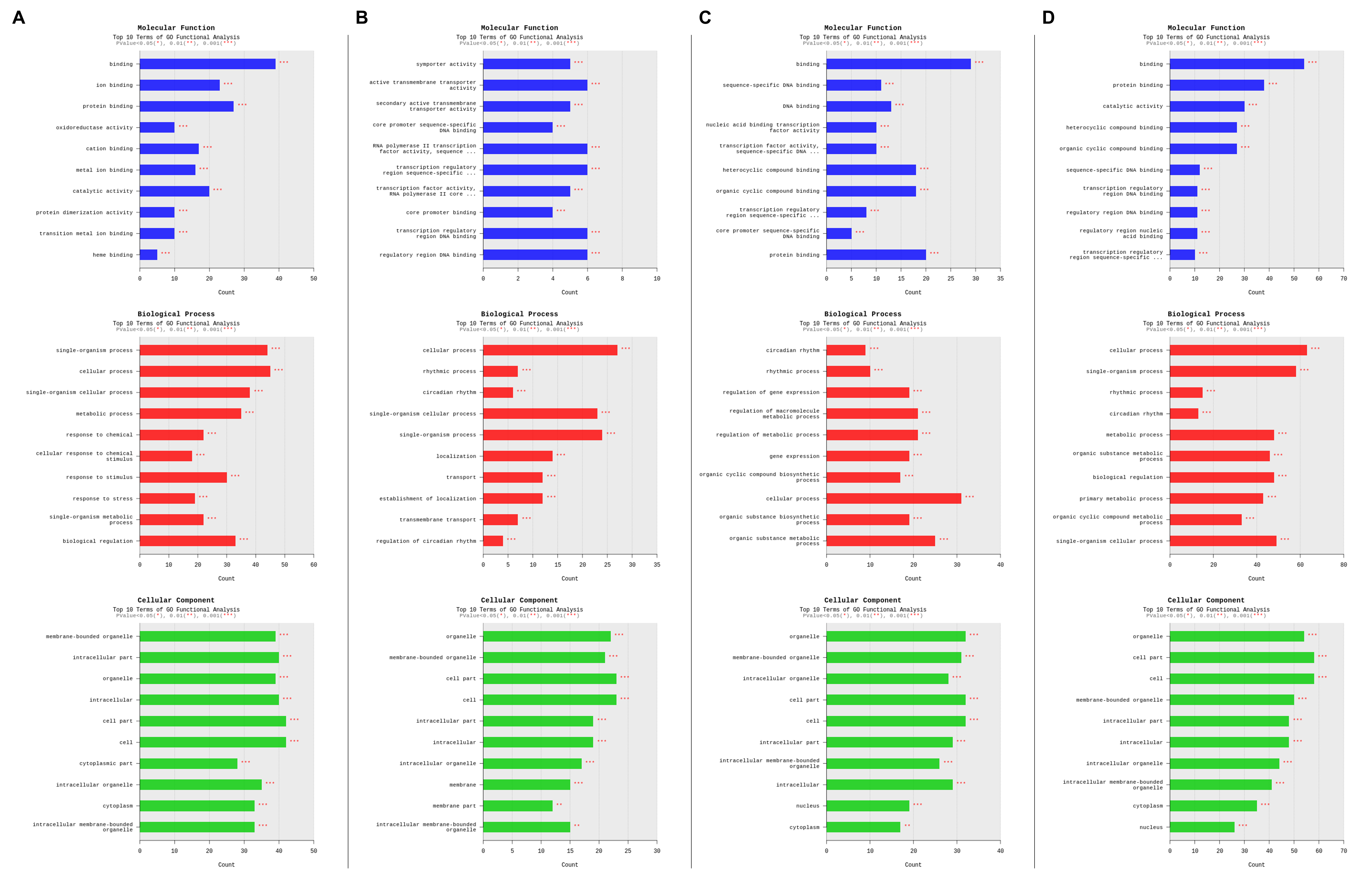


Figure S6. Gene ontology (GO) analysis results of ileal transcriptomes of (A) the ampicillin-induced gut dysbiosis group, (B) *Lactobacillus casei* ATG-F1-treated group, (C) *L. reuteri* ATG-F3-treated group, and (D) *L. reuteri* ATG-F4-treated group. Top 10 terms of GO functional analysis results of molecular function, biological process, and cellular components are shown. The image is in high resolution and can be zoomed in for detailed legends in this supplementary material. Statistical significance is as follows: ***p* < 0.01 and ****p* < 0.001 compared to the control group.


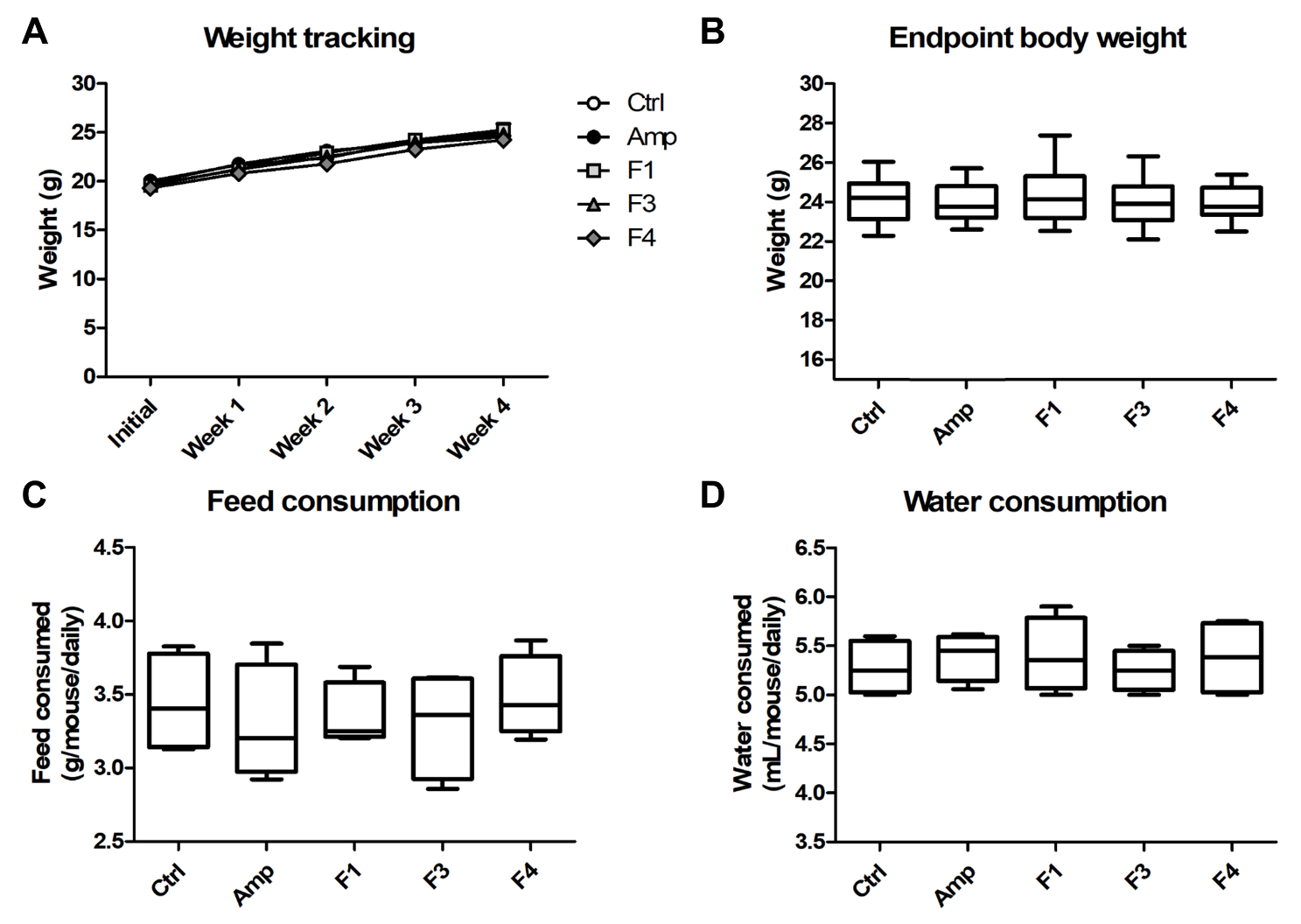


Figure S7. Physiological monitoring of each experimental groups. Experimental groups are as follows: control group (Ctrl), ampicillin-induced gut dysbiosis group (Amp), *Lactobacillus casei* ATG-F1 (F1)-treated, *L. reuteri* ATG-F3 (F3)-treated, and *L. reuteri* ATG-F4 (F4)-treated mice. No significant differences were observed in each measured factor.

**
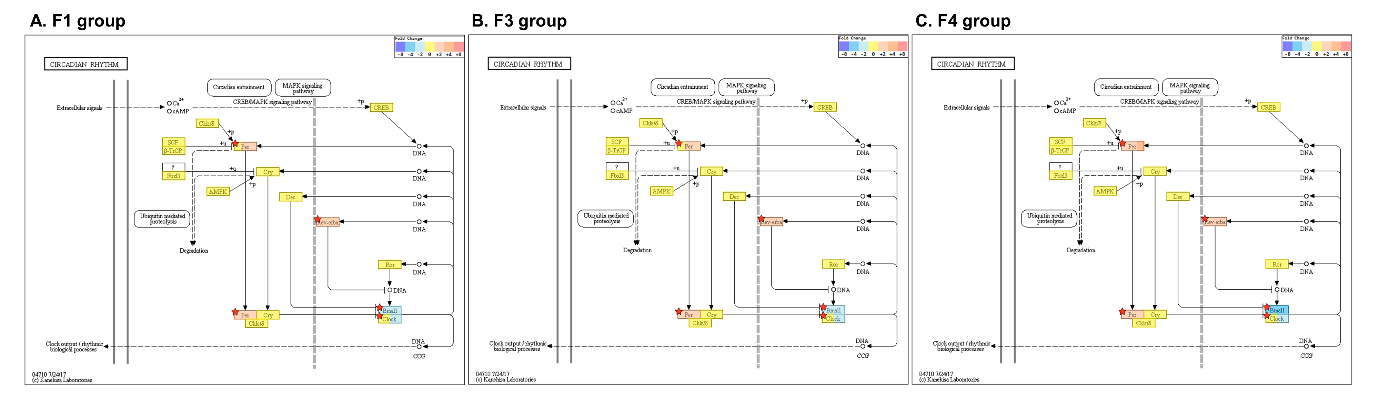
**

Figure S8. The circadian rhythm KEGG pathway maps (map04710) of gene expression in the ileum samples of mice treated with *Lactobacillus casei* ATG-F1 (F1), *L. reuteri* ATG-F3 (F3), and *L. reuteri* ATG-F4 (F4). Red stars indicate statistical significance at *p* < 0.05.
